# Optic nerve head factors associated with initial central visual field defect in primary open-angle glaucoma

**DOI:** 10.1038/s41598-024-58749-6

**Published:** 2024-04-05

**Authors:** Eunoo Bak, Martha Kim, Seok Hwan Kim, Kyoung Min Lee

**Affiliations:** 1 Department of Ophthalmology, Uijeongbu Eulji Medical Center, Eulji University School of Medicine, Uijeongbu, Republic of Korea; 2 Department of Ophthalmology, Seoul National University College of Medicine, Seoul, Republic of Korea; 3 Department of Ophthalmology, Dongguk University Ilsan Hospital, Goyang, Korea; 4 The One Seoul Eye Clinic, Seoul, Korea; 5 Department of Ophthalmology, Seoul National University Boramae Medical Center, 39 Boramae Road, Dongjak-gu, Seoul, 07061 Korea

**Keywords:** Optic nerve diseases, Risk factors

## Abstract

We investigated optic nerve head factors associated with initial parafoveal scotoma (IPFS) in primary open-angle glaucoma. Eighty (80) patients with an IPFS and 84 patients with an initial nasal step (INS) were compared. Central retinal vascular trunk (CRVT) deviation from the Bruch’s membrane opening (BMO) center was measured as a surrogate of lamina cribrosa (LC)/BMO offset, and its obliqueness was defined as the absolute value of angular deviation from the fovea-BMO axis. Proximity of retinal nerve fiber layer defect (RNFLD) was defined as the angular deviation of the inner RNFLD margin from the fovea-BMO axis. Microvasculature dropout (MvD) was defined as a focal sectoral capillary dropout with no visible microvascular network identified in the choroidal layer. Factors associated with IPFS, as compared with INS, were assessed using logistic regression analyses and conditional inference tree analysis. The IPFS group had more oblique CRVT offset (*P* < 0.001), RNFLD closer to the fovea (*P* < 0.001), more MvD (*P* < 0.001), and more LC defects (*P* < 0.001) compared to the INS group. In logistic regression analyses, obliqueness of CRVT offset (*P* = 0.002), RNFLD proximity (*P* < 0.001), and MvD (*P* = 0.001) were significant factors influencing the presence of IPFS. Conditional inference tree analysis showed that RNFLD closer to the fovea (*P* < 0.001) in the upper level, more oblique CRVT offset (*P* = 0.013) and presence of MvD (*P* = 0.001) in the lower level were associated with the probability of having IPFS. IPFS was associated with closer RNFLD location to the fovea when assessed from the BMO. Oblique LC/BMO offset may not only mask RNFLD proximity to the fovea due to a deviated funduscopic disc appearance, but also potentiate IPFS via focal LC defect and MvD.

## Introduction

Although glaucoma has been considered to affect midperipheral visual function in its early stages and to gradually progress to loss of central visual function until late stages of the disease process, some patients suffered the central visual field defects even in their early stages of glaucoma^1^. Fixation threatening parafoveal scotoma (PFS) is of concern, as fixation is strongly associated with the activities of daily living that determine the quality of life^2^. The association of PFS with normal or even low intraocular pressure (IOP)^3^ and with systemic risk factors such as systemic hypotension, migraine, Raynaud’s phenomenon, and sleep apnea^4^ might suggest the role of a vascular mechanism.

The initial site of visual field defect, however, also has been reported to be associated with the optic nerve head (ONH) structure. PFS has been more frequently found in eyes with focal lamina cribrosa (LC) defects^5^, large disc-foveal angle^6^, and microvasculature dropout (MvD)^7^. Furthermore, visual field defect location is known to be affected by the location of central retinal vascular trunk (CRVT): non-centered CRVT has been reported to be associated with more rapid central visual field loss^8^, and vertical position of the CRVT affected visual field defect location^9^.

Among these, CRVT deviation is thought to represent the underlying LC offset from the Bruch’s membrane opening (BMO). In contrast to LC depth or curvature, which can be referenced for evaluation of LC deviation in the axial direction along the *z*-axis, the LC/BMO offset reflects LC deviation from the ONH canal in the *x–y* plane. In one study, the LC/BMO offset was larger in the glaucomatous eyes of unilateral glaucoma patients^10^, and in others, was associated with the initial hemisphere of glaucomatous defect in various kinds of glaucoma^11–13^, which is suggestive of ONH structural vulnerability to glaucomatous defect acquired during eyeball expansion. The presence of LC/BMO offset is not only problematic when assessing retinal nerve fiber layer defect (RNFLD) proximity with a funduscopic optic disc as a reference point^14^, but is also known to be associated with focal LC defects^11^ and MvD^15^, which are known risk factors of PFS. To the best of our knowledge, there has been no study evaluating PFS risk factors relative to LC/BMO offset. The purpose of this study, then, was to identify and evaluate the association of ONH factors, including LC/BMO offset, with initial parafoveal scotoma (IPFS).

## Results

In total, 182 primary open-angle glaucoma (POAG) eyes that had undergone an optical coherence tomography angiography (OCT-A) examination and had an isolated IPFS or initial nasal step (INS) were included. Of these eyes, 18 were excluded because of poor-quality OCT-A images. Of the remaining 164 eyes, 80 had IPFS and 84 had INS (normal-tension glaucoma: 71 eyes with IPFS and 76 eyes with INS). In the IPFS group, papillomacular bundle defect was found in 3 cases (3.7%).

The clinical characteristics of the POAG patients with IPFS and those with INS are compared in Table 1. The IPFS group showed a more oblique CRVT offset (*P* < 0.001) and closer proximity of RNFLD (*P* < 0.001) than the INS group. MvD was observed in 36 of 80 eyes (45.0%) in the IPFS group, but in only 9 of 84 eyes (10.7%) in the INS group (*P* < 0.001). There was excellent interobserver agreement regarding MvD detection (k = 0.828). Focal LC defect was observed in 42 of 80 eyes (52.5%) in the IPFS group but in only 17 of 84 eyes (20.2%) in the INS group (*P* < 0.001). The inter-observer ICC for CRVT deviation measurement was 0.96 (95% CIs 0.95–0.98). This value indicated excellent agreement for the measurement^16^.

**Table 1:** Demographic data according to initial location of visual field defect.

	IPFS (n = 80)	INS (n = 84)	*P*-value
Age	61.1 ± 12.5	58.1 ± 12.6	0.10*
Male	34 (42.5%)	46 (54.8%)	0.16^†^
IOP (mmHg)	16.5 ± 4.2	16.4 ± 3.5	0.86*
Axial length (mm)	24.45 ± 1.32	24.85 ± 1.65	0.10*
Average RNFL thickness (μm)	75.5 ± 12.8	76.2 ± 12.3	0.73*
MD (dB)	–3.00 ± 1.79	–3.53 ± 2.38	0.33*
PSD (dB)	4.46 ± 2.41	4.22 ± 2.19	0.74*
β-zone PPA	77 (96.3%)	79 (94.0%)	0.51^†^
Offset index	0.39 ± 0.23	0.45 ± 0.23	0.14*
Obliqueness of CRVT offset (°)	37.61 ± 24.80	16.31 ± 14.76	< 0.001*
Proximity of RNFLD (°)	31.72 ± 10.27	46.94 ± 12.82	< 0.001*
MvD	36 (45.0%)	9 (10.7%)	< 0.001^†^
Focal LC defect	42 (52.5%)	17 (20.2%)	< 0.001^†^

Values are mean (SD) and n (%).

*IPFS* initial parafoveal scotoma, *INS* initial nasal step, *IOP* intraocular pressure, *RNFL* retinal nerve fiber layer, *MD* mean deviation, *PSD* pattern standard deviation, *dB* decibels, *PPA* parapapillary atrophy, *CRVT* central retinal vascular trunk, *RNFLD* RNFL defect, *MvD* microvasculature dropout, *LC* lamina cribrosa.

*Comparison performed using independent *t*-test.

^†^Comparison performed using Chi-square test.

The factors associated with presence of IPFS were determined by logistic regression analyses (Table 2). In the univariate analysis, more oblique CRVT offset (*P* < 0.001), closer proximity of RNFLD (*P* < 0.001), presence of MvD (*P* < 0.001), presence of focal LC defect (*P* < 0.001) were associated with presence of IPFS. Multivariate analyses were performed in 2 ways to account for multicollinearity between MvD and focal LC defect. The analyses revealed that more oblique CRVT offset (*P* = 0.002), closer proximity of RNFLD (*P* < 0.001), presence of MvD (*P* = 0.001) were significant factors affecting presence of IPFS.

**Table 2:** Factors associated with presence of initial parafoveal scotoma.

Variable	Univariable analysis			Multivariable analysis 1*			Multivariable Analysis 2*		
	Odds ratio	95% CI	*P*-value	Odds ratio	95% CI	*P*-value	Odds ratio	95% CI	*P*-value
Age, per 1 year older	1.020	0.995–1.046	0.12						
Male (vs. female)	1.561	0.843–2.892	0.16						
IOP, per 1 mmHg larger	1.007	0.930–1.092	0.86						
Axial length, per 1 mm longer	0.835	0.677–1.030	0.09	1.020	0.775–1.342	0.89	1.001	0.769–1.303	0.99
Average RNFL thickness, per 1 μm thicker	0.997	0.971–1.020	0.73						
MD	1.129	0.973–1.311	0.11						
PSD	1.048	0.916–1.198	0.50						
β-zone PPA	1.624	0.375–7.033	0.52						
Offset index	0.351	0.092–1.330	0.12						
Obliqueness of CRVT offset, per 1° larger	1.059	1.036–1.083	** < 0.001**	1.038	1.013–1.063	**0.002**	1.035	1.010–1.060	**0.005**
Proximity of RNFLD, per 1° larger	0.896	0.898–0.950	** < 0.001**	0.935	0.906–0.966	** < 0.001**	0.935	0.906–0.964	**0.001**
MvD	6.818	3.004–15.477	** < 0.001**	4.880	1.840–12.944	**0.001**			
Focal LC defect	4.356	2.185–8.683	< 0.001				2.061	0.837–5.074	0.11

*CI* confidence interval, *IOP* intraocular pressure, *RNFL* retinal nerve fiber layer, *MD* mean deviation, *PSD* pattern standard deviation, *PPA* parapapillary atrophy, *CRVT* central retinal vascular trunk, *RNFLD* RNFL defect, *MvD* microvasculature dropout, *LC* lamina cribrosa. *Variables with P < 0.10 in the univariable analysis were included in the subsequent multivariable analysis. Statistically significant values are shown in bold.

In the univariate analysis investigating factors associated with presence of MvD, larger pattern standard deviation (PSD) (*P* = 0.040), more oblique CRVT offset (*P* < 0.001), closer proximity of RNFLD (*P* = 0.006), and presence of focal LC defect (*P* < 0.001) were associated with presence of MvD (Table 3). In the multivariate logistic regression analysis, more oblique CRVT offset (*P* = 0.016), closer proximity of RNFLD (*P* < 0.001), and presence of focal LC defect (*P* < 0.001) were associated with presence of MvD.

**Table 3:** Factors associated with presence of parapapillary choroidal microvasculature dropout.

Variable	Univariable analysis			Multivariable analysis*		
	Odds ratio	95% CI	*P*-value	Odds ratio	95% CI	*P*-value
Age, per 1 year older	1.022	0.993–1.051	0.14			
Male (vs. female)	1.229	0.617–2.448	0.56			
IOP, per 1 mmHg larger	1.040	0.954–1.135	0.37			
Axial length, per 1 mm longer	0.888	0.702–1.124	0.32			
Average RNFL thickness, per 1 μm thicker	0.984	0.957–1.011	0.24			
MD	0.954	0.814–1.118	0.56			
PSD	1.164	1.007–1.346	0.040	1.114	0.949–1.307	0.19
Offset index	0.790	0.182–3.439	0.75			
Obliqueness of CRVT offset, per 1° larger	1.029	1.013–1.046	** < 0.001**	1.020	1.004–1.036	**0.016**
Proximity of RNFLD, per 1° larger	0.968	0.945–0.990	**0.006**	0.938	0.908–0.969	**0.001**
Focal LC defect	9.837	4.460–21.694	** < 0.001**	9.316	4.086–21.243	** < 0.001**

*CI* confidence interval, *IOP* intraocular pressure, *RNFL* retinal nerve fiber layer, *MD* mean deviation, *PSD* pattern standard deviation, *CRVT* central retinal vascular trunk, *RNFLD* RNFL defect, *LC* lamina cribrosa.

*Variables with *P* < 0.10 in the univariable analysis were included in the subsequent multivariable analysis. Statistically significant values are shown in bold.

**Figure 1:**
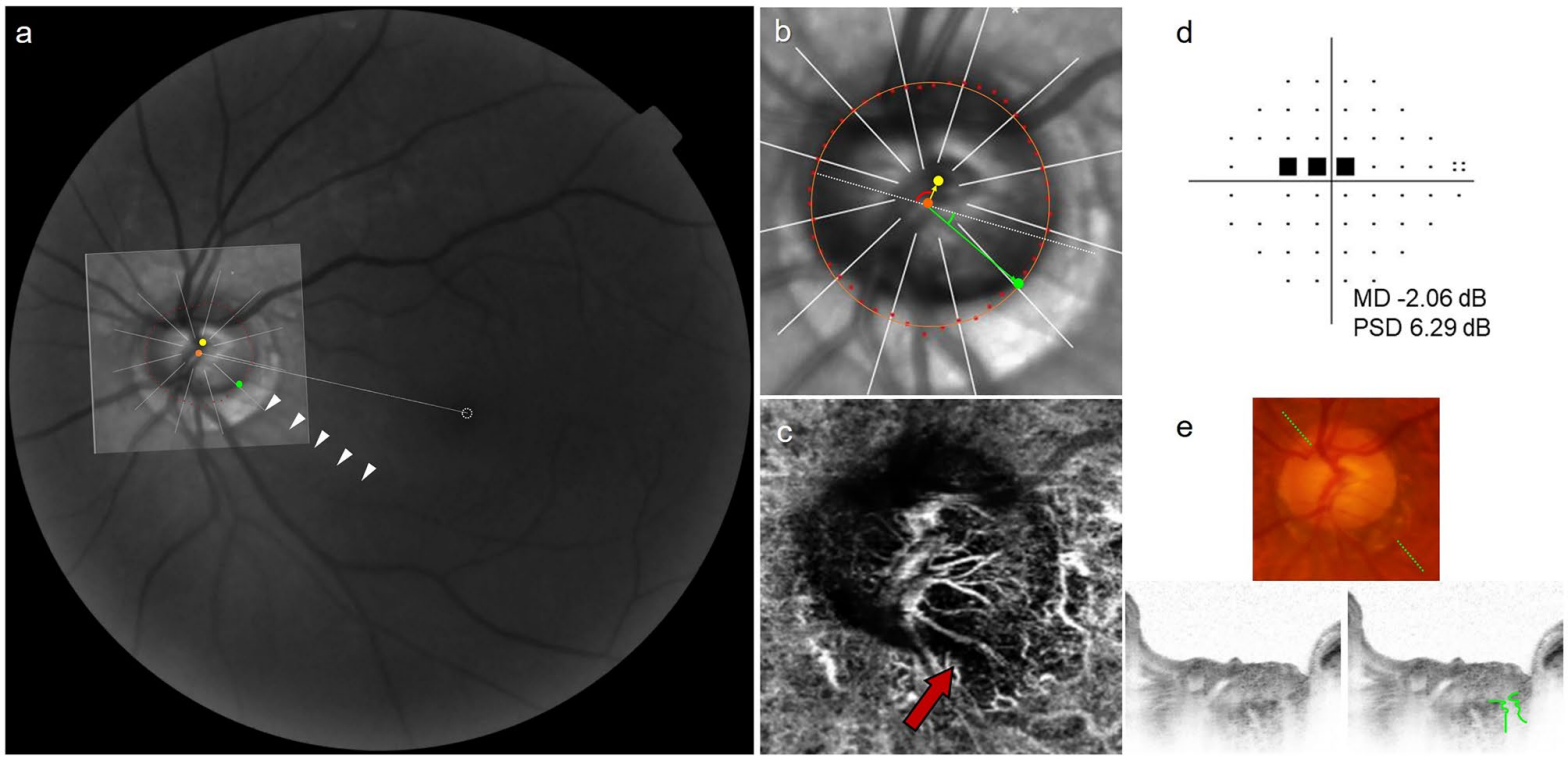
Representative case of eye with initial parafoveal scotoma (IPFS). (**a**) Red-free fundus photograph. The Bruch’s membrane opening (BMO, red dots), central retinal vascular trunk (CRVT) position (yellow dot), proximal margin of retinal nerve fiber layer defect (RNFLD, green dot), and fovea-BMO axis (white dotted line) are marked. RNFLD is demarcated with the white arrow head. (**b**) Infrared image obtained by OCT. Best-fitted ellipse for Bruch’s membrane opening (BMO, orange ellipse) is calculated with its center (orange dot). From the fovea-BMO axis (white dotted line), the obliqueness of CRVT (red angle) is 86.0°and the proximity of RNFLD (green angle) is 24.0°. (**c**) En face OCT angiography images obtained in choroidal layer. Microvasculature dropout (MvD) was marked with red arrow. (**d**) Humphrey visual field results showing IPFS. (**e**) Disc photograph and B-scan OCT image. The green dotted line indicates the location of the OCT scan. A B-scan OCT image (left) and the same image with the lines demarcated along the anterior surface of the lamina cribrosa (LC) with focal LC defect (right; green lines) are presented.

**Figure 2:**
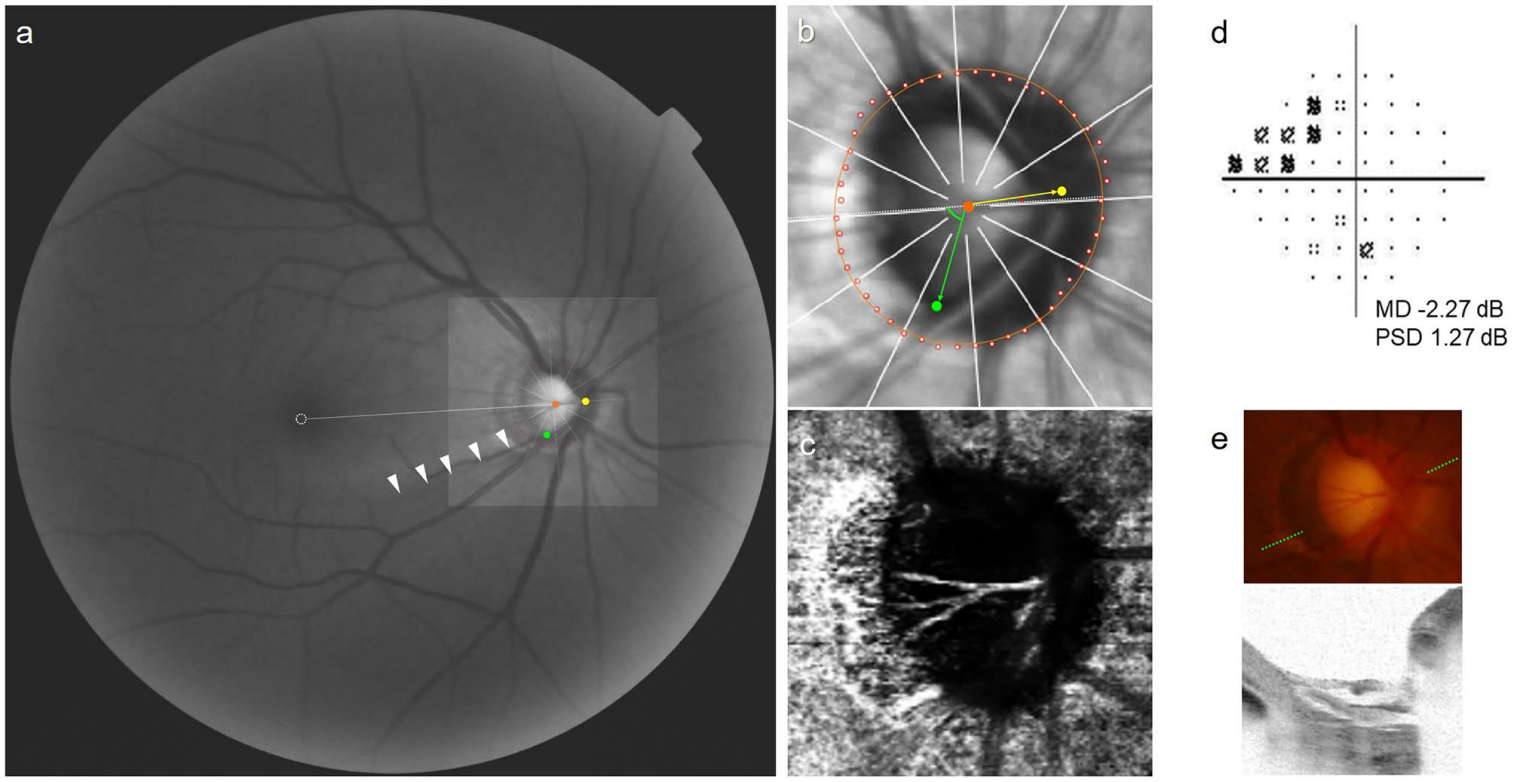
Representative case of eye with initial nasal step (INS). (**a**) Red-free fundus photograph. The Bruch’s membrane opening (BMO, red dots), central retinal vascular trunk (CRVT) position (yellow dot), proximal margin of retinal nerve fiber layer defect (RNFLD, green dot), and fovea-BMO axis (white dotted line) are marked. RNFLD is demarcated with the white arrow head. (**b**) Infrared image obtained by optical coherence tomography (OCT). Best-fitted ellipse for Bruch’s membrane opening (BMO, orange ellipse) is calculated with its center (orange dot). From the fovea-BMO axis (white dotted line), the obliqueness of CRVT (red angle) is 5.1° and the proximity of RNFLD (green angle) is 69.0°. (**c**) En face OCT angiography images obtained in choroidal layer. No microvasculature dropout (MvD) was detected. (**d**) Humphrey visual field results showing INS. (**e**) Disc photograph and B-scan OCT image. The green dotted line indicates the location of the OCT scan. No focal lamina cribrosa defect was detected.

Figure 1 shows an MvD eye of a patient in the IPFS group, and Fig. 2 shows a no-MvD eye of a patient in the INS group. The obliqueness of CRVT offset and the RNFLD proximity of the patient in the IPFS group were 86.0° and 24.0°, respectively. The respective values for the patient in the INS group were 5.1° and 69.0°. A focal LC defect was observed in a patient with IPFS who had an oblique CRVT offset and MvD.

**Figure 3:**
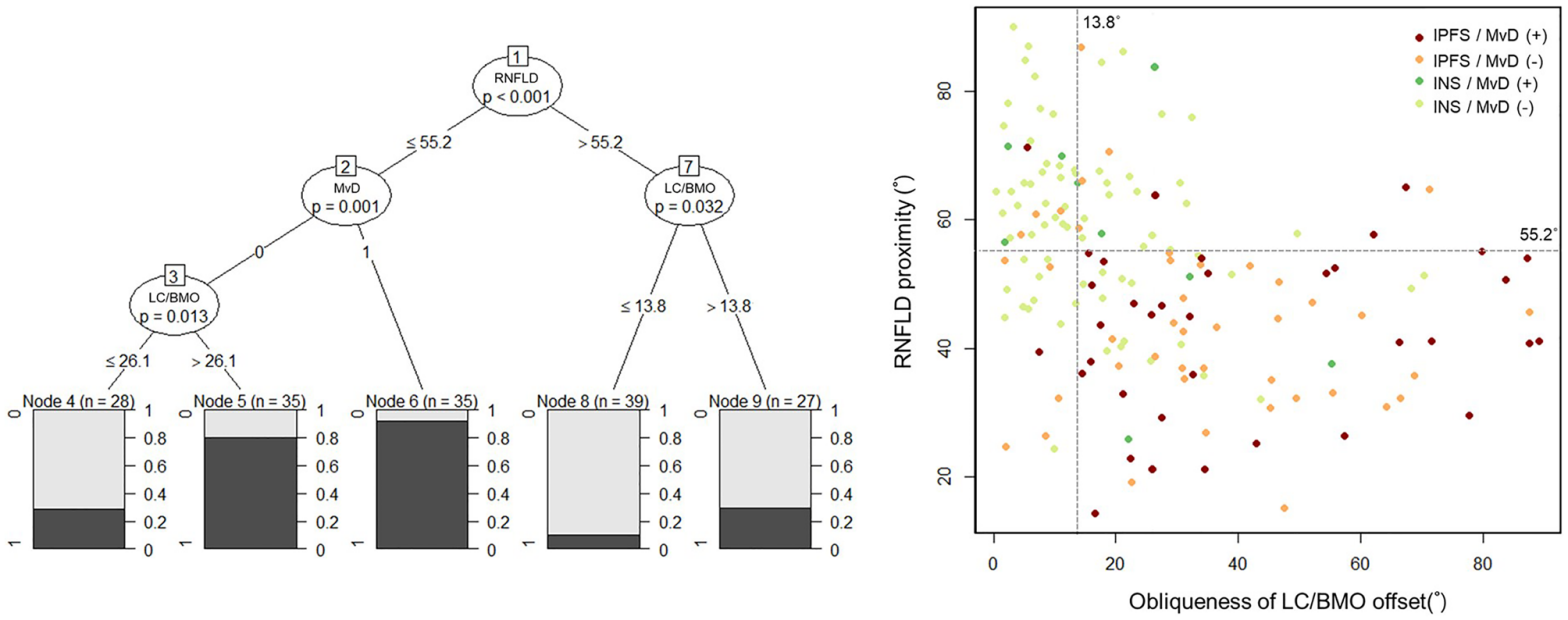
Conditional inference tree analysis. By a recursive partitioning method, this analysis allows for unbiased testing of both categorical and continuous variables without any statistical assumptions. By this analysis, the types of visual field defects were categorized into five terminal nodes. The probability of initial parafoveal scotoma (IPFS) in each node is demonstrated as a bar graph. First, the proximity of retinal nerve fiber layer defect (RNFLD) to the fovea was associated with IPFS in the upper level: IPFS was more frequent when RNFLD was within 55.2° of the fovea. Among those eyes, presence of microvasculature dropout (MvD) and oblique angular direction of offset between lamina cribrosa (LC) and Bruch’s membrane opening (BMO) were associated with higher probability of IPFS. In the eyes with RNFLD proximity over 55.2° from the fovea, more oblique LC/BMO offset was associated with higher probability of IPFS. In the scatter plot, subjects are classified as (1) IPFS with MvD (red dot), (2) IPFS without MvD (orange dot), (3) initial nasal scotoma (INS) with MvD (green dot), and (4) INS without MvD (light green dot). A black horizontal dotted line is drawn along 55.2° of RNFLD proximity and a black vertical dotted line is drawn along 13.8° of LC/BMO offset obliqueness to visualize the distribution of each group according to these two variables.

The conditional inference tree analysis revealed the hierarchy among the factors related to the presence of IPFS. First, RNFLD proximity was associated with IPFS in the upper level: IPFS occurred in closer proximity to RNFLD (i.e., closeness to the fovea). In the eyes with RNFLD closer to the fovea (≤ 55.2°), presence of MvD and more oblique CRVT/BMO offset were associated with higher frequency of IPFS occurrence. In the eyes with RNFLD farther away from the fovea (> 55.2°), more oblique CRVT/BMO offset was associated with higher frequency of IPFS occurrence (Fig. 3). For categories with CRVT/BMO offset > 13.8° and RNFLD ≤ 55.2° (n = 93), the rate of MvD presence was 36.5% (n = 34) and the overall probability of IPFS was 83.9% (n = 78).

## Discussion

In this study, we evaluated factors associated with patients having central scotoma in the early stage of glaucoma. The IPFS group had RNFLD closer to the fovea-BMO axis, higher incidence of MvD, and more oblique CRVT offset. MvD was associated with RNFLD closer to the fovea-BMO axis, more oblique CRVT offset and presence of focal LC defects. To the best of our knowledge, this is the first study to identify and confirm ONH factors associated with IPFS in glaucoma in consideration of LC/BMO offset.

Previous reports have presented several optic disc factors associated with IPFS, including disc-foveal angle^6^, disc-margin-to-fovea distance^17^, tilt ratio and optic disc rotation^18^. The optic disc, however, is not a distinct anatomic structure, but is a funduscopic silhouette representing the offset of openings in the ONH canal^19^. During eyeball growth, the retinal structure above the BMO is relatively preserved, while the underneath scleral structure is shifted^20,21^. Thus, the BMO might be a better reference point, rather than the funduscopic optic disc appearance which changes with growth, for assessment of RNFLD location. Recently, we reported that seemingly different RNFLD locations in myopic glaucoma eyes when assessed from the disc as a reference point were not different from those of non-myopic glaucoma eyes when assessed from the BMO as a reference point^14^. And in the present study, we showed that RNFLD location as assessed from the BMO is closely associated with the initial location of visual field defect.

Many studies have reported a clear spatial correlation between RNFLD and visual field defects^22,23^. RNFLDs near the fovea, such as papillomacular bundle defects, have been reported to be associated with decreased central visual field sensitivity^23,24^. Recently, however, Leung et al. reported that, having employed their new diagnostic modality, papillofoveal or papillomacular bundle defects were more common than expected^25^. This might explain some cases showing a poor structure–function relationship in the central area when assessed by conventional modalities^26^. From another perspective, in this study, we found that IPFS was associated with RNFLDs closer to the fovea when assessed from an unbiased reference point: the BMO.

The LC/BMO offset, however, might incur a confounding effect in determining of RNFLD location when the optic disc is used as a reference point. In this study, IPFS was associated with more oblique LC/BMO offset axis. From the optic disc perspective, IPFS patients have been reported to have a greater disc-foveal angle^6^ and a shorter disc-margin-to-fovea distance^17^. We speculated that more oblique LC/BMO offset could explain both the greater disc-foveal angle and the shorter disc-margin-to-fovea distance in those eyes by displacing the funduscopic optic disc location from the BMO (Supplementary Fig. S1). Oblique LC/BMO axis, moreover, renders the RNFLD location more peripheral relative to the optic disc (Supplementary Fig. S1). Therefore, clinicians who do not consider LC/BMO offset might be confused as to why some patients have IPFS with a seemingly similar RNFLD location relative to the foveal-disc axis. In this light, we speculated that by adopting the BMO rather than funduscopic optic disc as a reference point, many parameters such as angular location of RNFLD or macular vulnerability zone could be modified to better represent values.

Besides the confounding effect, the LC/BMO offset might reflect hidden structural changes in the ONH canal such as focal LC defects or MvD. Sawada et al. reported that in myopic eyes, focal LC defects might develop on the temporal side of the LC^27^, resulting in more central scotomas in cases where the LC/BMO offset is large^18,24^. In another study, more oblique LC/BMO offset was reported to have more focal LC defects^11^, which were associated with MvD in previous studies^15,28^. Han et al. proposed the relationship between focal LC defect and MvD influenced by the structural vulnerability at the location of border tissue^15^. We speculated that not only the location of externally oblique border tissue but also the location of CRVT suggests the presence of LC/BMO offset, which reflects tensile stress over the LC. With this change, variations in the pressure induced strains to the LC structure, which may contribute to create regional difference in LC damage, incurring structural vulnerability^9^ that results in focal LC defects and MvD.

In the present study, MvD was associated with IPFS, in line with previous investigations^7,29^. Presence of MvD has been reported to be associated with more progressive and severe glaucoma^30,31^. We speculated that MvD would represent a more hostile environment for the RGC axons regardless of whether it is the cause or the result of glaucomatous damage. Compressive and tensile stresses across the LC not only induce mechanical injury but also may cause circulatory impairment and oxygenation failure^32^. Thus, regional poor perfusion, detected by the presence of MvD, might potentiate further injury, such as RGC axons may lose structural and metabolic support, leading to the loss of RGCs. If oxygenation does matter, it would be more toxic close to the fovea as it would require a higher metabolic demand. The association of IPFS with vascular risk factors support this speculation^4,33^.

This study has several limitations. First, all of the participants were South Korean, and probable ethnic differences might be both pertinent and significant. Second, selection bias may have been incurred. Only early-glaucoma patients with isolated IPFS or INS were included, thus the results might not be applicable to advanced-stage glaucoma. Further, cases with multiple papillomacular bundle defects, which are observed in highly myopic eyes^24^, might have been excluded. That would be one reason why IPFS were observed more frequently in cases with oblique LC/BMO offset. Cases with non-oblique LC/BMO offset might have multiple RNFLDs and not have isolated IPFS or INS. Comparing these distinct patterns of VF defect in the early stages, however, may help find pathogenic differences between IPFS and INS. Additionally, parafoveal VF defect are functionally important in terms of location because they have a greater impact on vision and reading compared to peripheral VF defect^34^. Therefore, ONH structural features for having high risk of IPFS is important clinically. Third and lastly, we could not deduce any causal relationship for central visual field defect other than RNFLD location. What we did find was only an association among oblique LC/BMO offset, focal LC defect, and MvD. Further study is required to identify which factor induces the others.

In conclusion, central visual field defect developed even in the early stages of glaucoma in patients with closer RNFLD location to the fovea, more oblique LC/BMO offset, and MvD. LC/BMO offset not only induces a seemingly more remote RNFLD location from the disc perspective, but also potentiates IPFS via focal LC defect and MvD. The LC/BMO offset, therefore, should be taken into consideration both in assessing the location of glaucomatous damage and in evaluating underlying ONH anatomy. Patients with oblique LC/BMO offset and MvD should be monitored closely, since they may be more at risk of functional disability due to central scotoma, even in the early stages of glaucoma.

## Methods

### Study subjects

This study was based on POAG patients already enrolled in the Boramae Glaucoma Imaging Study, an ongoing prospective study at Seoul National University Boramae Medical Center (Seoul, Korea)^11,12,35^. Written informed consent to participate was provided by all. The study protocol was approved by the Seoul National University Boramae Medical Center Institutional Review Board (IRB no. 30-2017-33) and conformed to the tenets of the Declaration of Helsinki.

All of the participants underwent comprehensive ophthalmic examinations that included best-corrected visual acuity (BCVA), Goldmann applanation tonometry, a refraction test, slit-lamp biomicroscopy, gonioscopy, disc photography and red-free fundus photography (TRC-NW8; Topcon, Tokyo, Japan), axial length measurement (IOL Master version 5; Carl Zeiss Meditec, Dublin, CA, USA), spectral-domain OCT/OCT -A (Spectralis OCT; Heidelberg Engineering, Heidelberg, Germany), and standard automated perimetry (Humphrey Field Analyzer II 750, 24-2 Swedish Interactive Threshold Algorithm; Carl Zeiss Meditec)^11,12,35^. The circumpapillary retinal nerve fiber layer (RNFL) thickness was measured using spectral-domain OCT (Spectralis). The magnification error was corrected by entering the corneal curvature of each eye into the OCT system before scanning. While acquiring OCT images, subjects were asked to fixate on a target, and images were acquired with their forehead and chin fixed on the headrest^19^. Before the initiation of treatment, IOP was measured repeatedly (typically 5 times) on the same or different days. Among the documented IOP readings for each subject, the highest IOP was used in the subsequent analysis.

Glaucomatous optic nerve damage was defined by rim thinning, notching, and the presence of RNFLD, and was evaluated by two glaucoma specialists (SHK and KML). POAG was defined as glaucomatous optic nerve damage and associated visual field defects with an open iridocorneal angle. Glaucomatous visual field defect was defined as (1) outside-normal-limits glaucoma hemifield test results or (2) three abnormal points, with a *P*-value of less than 5% probability of being normal and one with a *P*-value of less than 1% by pattern deviation or (3) PSD of less than 5%. Visual field defects were confirmed on 2 consecutive reliable tests (fixation loss rate of ≤ 20%, false-positive and false negative error rates of ≤ 25%).

The inclusion criterion was POAG patients having only an isolated IPFS or INS in one hemifield, as defined below. The exclusion criteria were eyes with a BCVA worse than 20/40; a spherical equivalent less than − 8.0 or more than + 3.0 diopters; a history of intraocular surgery, with the exception of uneventful cataract surgery; a history of uveitis or inflammatory disease; any retinal or neurologic diseases; RNFLD margin could not be determined clearly; poor-quality image (i.e., quality score < 15) of any section on OCT radial scans; incomplete demarcation of BMO margin, and cases where the CRVT was located within the BMO but was impossible to determine clearly due to vessel bifurcation. If both eyes of a patient were eligible, the eye with the better visual field mean deviation value was selected as the study eye.

### Visual field criteria for IPFS and INS

**Figure 4:**
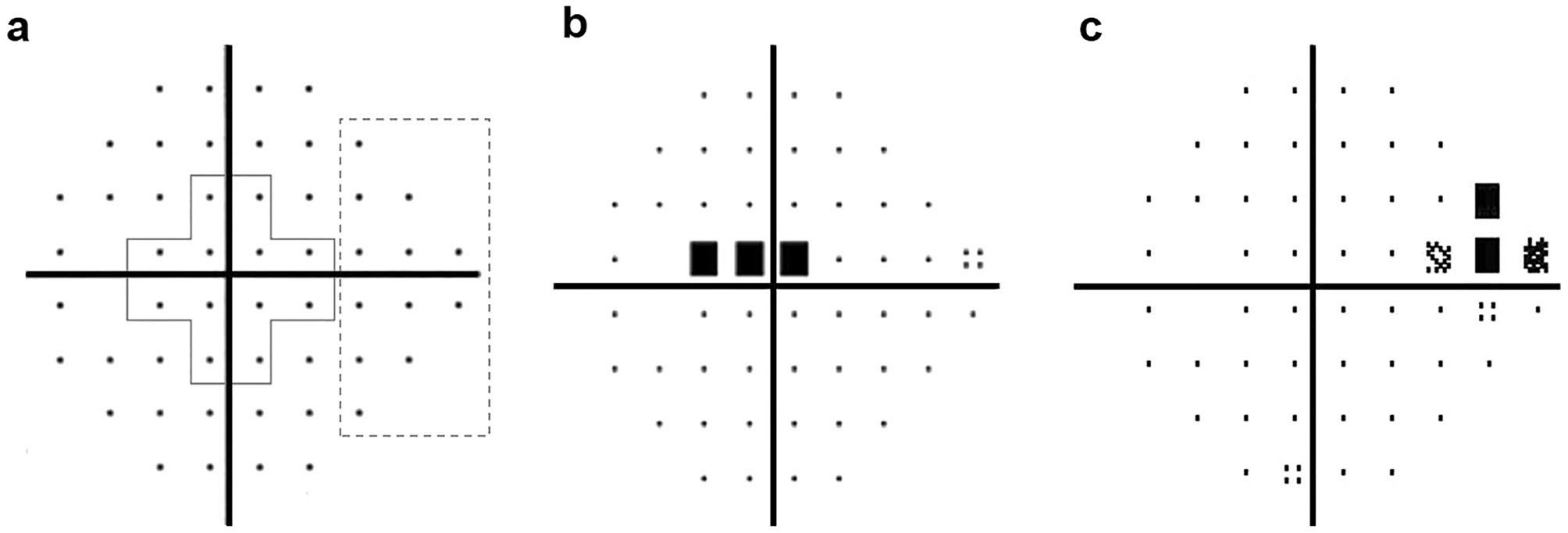
Two subfields of the pattern deviation plot. (**a**) An initial parafoveal scotoma (IPFS) has abnormal points within 12 points of a central 10° of fixation (solid box) in 1 hemifield, and no abnormalities within the 12 nasal periphery points (dashed box). The initial nasal step (INS) has abnormal points within 12 nasal peripheral points (dashed box) in 1 hemifield and no abnormalities within the central 10° (solid box). (**b**) Representative image of IPFS. (**c**) Representative image of INS.

The initial location of visual field defects was categorized as IPFS or INS, based on criteria specified elsewhere (Fig. 4)^4,36^. Based on all of the obtained pattern deviation plots for each subject, the locations of the three connected abnormal points with a *P*-value of less than 5% probability of being normal and at least one with a *P*-value of less than 1% appearing repetitively were determined: (1) within 12 points of the central 10° of fixation restricted to one hemisphere: IPFS, and (2) in the nasal periphery outside 10° of fixation restricted to one hemisphere: INS. Abnormal points outside the demarcated lines did not affect the categorization unless their presence satisfied both the IPFS and INS criteria. In those cases, they were excluded from the analysis.

### Assessment of LC/BMO offset using CRVT

The peripapillary area was imaged by OCT using the enhanced depth imaging (EDI) technique. The BMO was demarcated on infrared images obtained by the Glaucoma Module Premium Edition of Spectralis OCT. In this mode, the deep-ONH complex was imaged with 24 high-resolution radial scans taken in 15° increments. Each radial scan image was obtained by averaging 24 individual B-scan images, with OCT automatically detecting the BMO margin. The BMO margin was reviewed and errors were corrected manually by one glaucoma specialist (EB). Based on the edited BMO margin, the Spectralis machine calculated the area and center of the BMO and determined the fovea-BMO axis.

**Figure 5:**
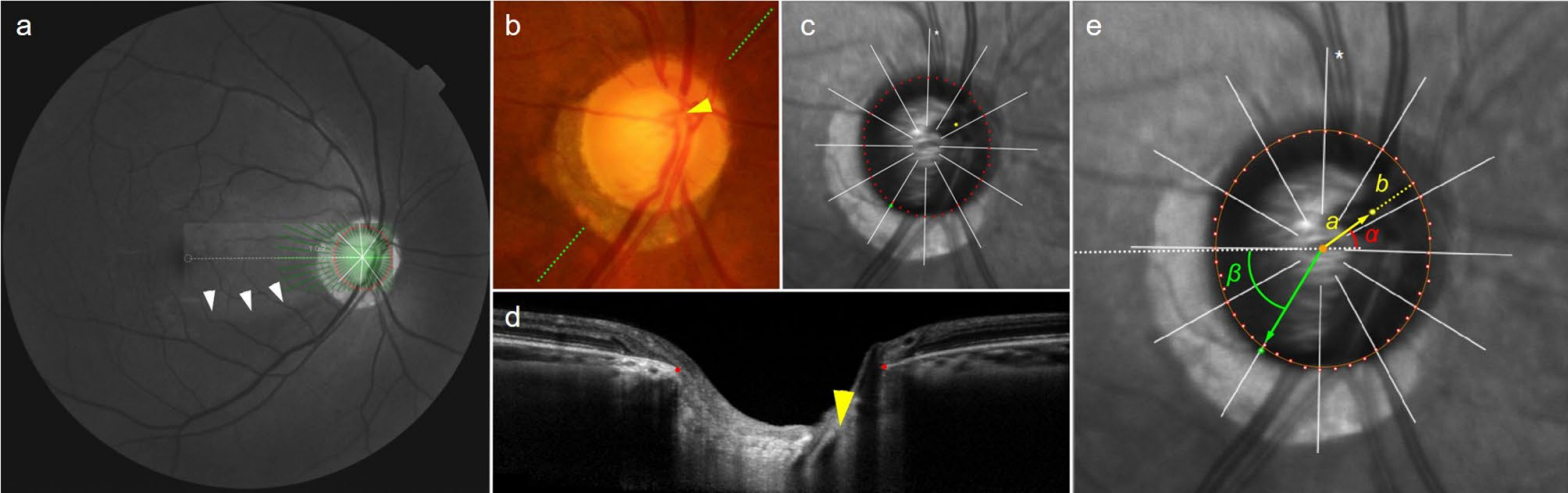
Measurements of central retinal vascular trunk (CRVT) deviation and proximity of retinal nerve fiber layer defect (RNFLD) from Bruch’s membrane opening (BMO) center. (**a**) Red-free fundus photo shows inferior RNFLD (white arrowheads). An infrared image obtained by OCT is transposed to show the BMO margin (red dots) and the fovea-BMO axis (white dotted line). (**b**) Disc photograph. The green dotted line indicates the location of the OCT scan. The yellow arrowhead indicates the location of the CRVT. (**c**) Infrared image of peripapillary area obtained by OCT. The BMO (red dots), CRVT position (yellow dot) and the proximal margin of the RNFLD (green dot) are marked. (**d**) B-scan OCT image. The red dots indicate the BMO margin. The emergence of the CRVT (yellow arrowhead) from the lamina cribrosa is clearly visible. (**e**) Calculation of angular deviations. Best-fitted ellipse for BMO (orange ellipse) is calculated with its center (orange dot). The fovea-BMO axis is set as the reference line (white dotted line). The ‘obliqueness of CRVT’ is measured as the angular deviation of CRVT from the nasal side of the reference line (α, red angle). The positive value indicates a superiorly deviated CRVT, and the negative value indicates an inferiorly deviated CRVT. The offset index is defined as the ratio of the distance from the BMO center to the location of the CRVT (*a*) and the distance from the BMO center to the BMO margin (*b*) in the same CRVT direction. The ‘proximity of RNFLD’ is measured as the angular deviation between the inner RNFLD margin and the temporal side of the reference line (β, green angle).

The CRVT position, as indicated previously, was measured from the BMO center (Fig. 5)^11,12,19^. Its emergence was demarcated on fundoscopic infrared images and color-disc photography, and was confirmed by cross-sectional OCT imaging in all cases. In cases of CRVT invisibility, OCT-A (Spectralis) was used to determine the presence of the CRVT within the BMO. CRVT offset was recorded as the (1) angular deviation from the BMO center and (2) its extent^11,12,19^. Angular deviation was measured based on the right-eye orientation, with the fovea-BMO axis at 0° (a positive value indicative of superior location, and a negative value, inferior location). Obliqueness of CRVT offset was defined as the absolute value of angular deviation in the quadrant where the CRVT was located, in order to represent the extent to which the CRVT deviated from the vertical axis^12^. The extent, defined as the “offset index”, was measured and calculated as the distance of the CRVT from the BMO center (*a*) divided by the distance of the BMO margin from the BMO center in that direction (*b*)^11,12^. To calculate these parameters, the CRVT positions were marked on infrared images, and their pixel values were read by our customized software^19^. In cases of CRVT invisibility, the offset index was defined as 1.0, and the angular deviation was not determined. Such cases were excluded for further analysis of the angular deviation of CRVT offset.

### Assessment of RNFLD proximity to fovea

The location of RNFLD was determined in terms of its proximity to the fovea. The inner RNFLD margin, which was closer to the fovea, was marked on red-free fundus photographs. Red-free fundus photographs were superimposed and aligned with infrared fundus images using a commercially available software (Photoshop; Adobe, San Jose, CA, USA). From the BMO center and using the fovea-BMO axis as a reference line, the angular deviation was measured to the point where the inner defect margin crossed the BMO (Fig. 5). As was done for the CRVT, the angular deviation was measured based on the right-eye orientation, with the fovea-BMO axis at 0° (a positive value indicative of superior location, and a negative value, inferior location). Proximity of the inner RNFLD margin was defined as the absolute value of angular deviation in the quadrant where the inner RNFLD margin was located^12^. To calculate these parameters, the crossing points of the inner RNFLD margin with the BMO were marked on infrared images and their pixel values were read by our customized software^11,12,19^.

In the IFPS group, papillomacular bundle defect was evaluated. The papillomacular bundle area was established with reference to the relevant literature^37,38^. The temporal region of the optic disc was divided evenly into six sectors of 30° based on a reference line connecting the optic disc and foveal center. Then, the angular location within the − 30.0 to  + 30.0° sectors was deemed to be the papillomacular bundle area, and papillomacular bundle defect was confirmed when the proximal border of RNFLD was within this area.

### Determination of parapapillary choroidal MvD

The ONH and peripapillary area were imaged using a commercially available OCT-A device (Spectralis; Heidelberg) with a central wavelength of 880 nm, an acquisition speed of 85 kHz, and lateral and axial resolutions of 5.7 and 3.9 µm per pixel, respectively. ^1^Scans were obtained in a 20° $\times$ 20° pattern consisting of 512 clusters of 5 repeated B-scans centered on the optic disc. The parapapillary choroidal microvasculature was evaluated on en-face images of the parapapillary deep layer, generated based on automated layer segmentation performed by the OCT instrument software. The en-face images of the deep layer were derived from an en-face slab, extending from the retinal pigment epithelium to 390 µm below Bruch’s membrane, which was sufficient to include the full thickness of the choroid and the inner scleral surface, as reported previously^7^.

The MvD was defined on the deep layer en-face images as a focal sectoral capillary dropout with no visible microvascular network identifiable^28,39,40^. When the circumferential width of the area with capillary dropout appeared to be more than one half clock hour of the BMO circumference, it was considered a disruption of the microvascular network and was deemed an MvD (Fig. 1c)^7^. Two independent observers (EB and KML) identified MvDs while blinded to the clinical data of the participants. Disagreements were resolved by a third adjudicator (SHK).

### Assessment of focal LC defect

The EDI OCT images were carefully reviewed for focal LC defects violating the smooth curvilinear U- or W-shaped contour^41^. This was done by one glaucoma specialist (EB) masked to participants’ clinical information such as the infrared optic disc photography acquired by the OCT device. A focal LC defect was required to exhibit a diameter of 100 µm or more and a depth of 30 µm or more on cross-sectional OCT images^41–43^. To avoid false-positives, a focal LC defect detected was required to have at least 1 additional adjacent OCT image for which findings were similar.

### Data analysis

The intergroup characteristics were compared by independent samples *t*-test for normally distributed data and chi-squared testing for continuous and categorical variables. Interobserver agreement confirming the presence of MvD was assessed using kappa statistics (k values). To determine the inter-observer agreement on the CRVT deviation measurement, the intra-class correlation coefficient (ICC) with its CI was calculated by two independent examiners (EB and KML). Factors influencing the presence of IPFS and MvD were assessed using logistic regression analyses. Variables with *P*-values of less than 0.1 in the univariate analysis were included in the subsequent multivariate analysis. Conditional inference tree analysis was used to determine the hierarchy of risk factors. All of the statistical analyses were performed using SPSS software version 21.0 (SPSS, Inc., Chicago, IL, USA) and R statistical packages version 3.4.3. A *P*-value of less than 0.05 was considered to represent statistical significance.

### Supplementary Information

Supplementary Figure 1.

Supplementary Legends.

## Data Availability

The datasets used and/or analysed during the current study available from the corresponding author on reasonable request.
