# Supplementary figures and images for: Optic nerve head factors associated with initial central visual field defect in primary open-angle glaucoma
Source: Sci Rep. 2024 Apr 5;14:8000. doi: 10.1038/s41598-024-58749-6 (PMC10997601; doi:10.1038/s41598-024-58749-6)

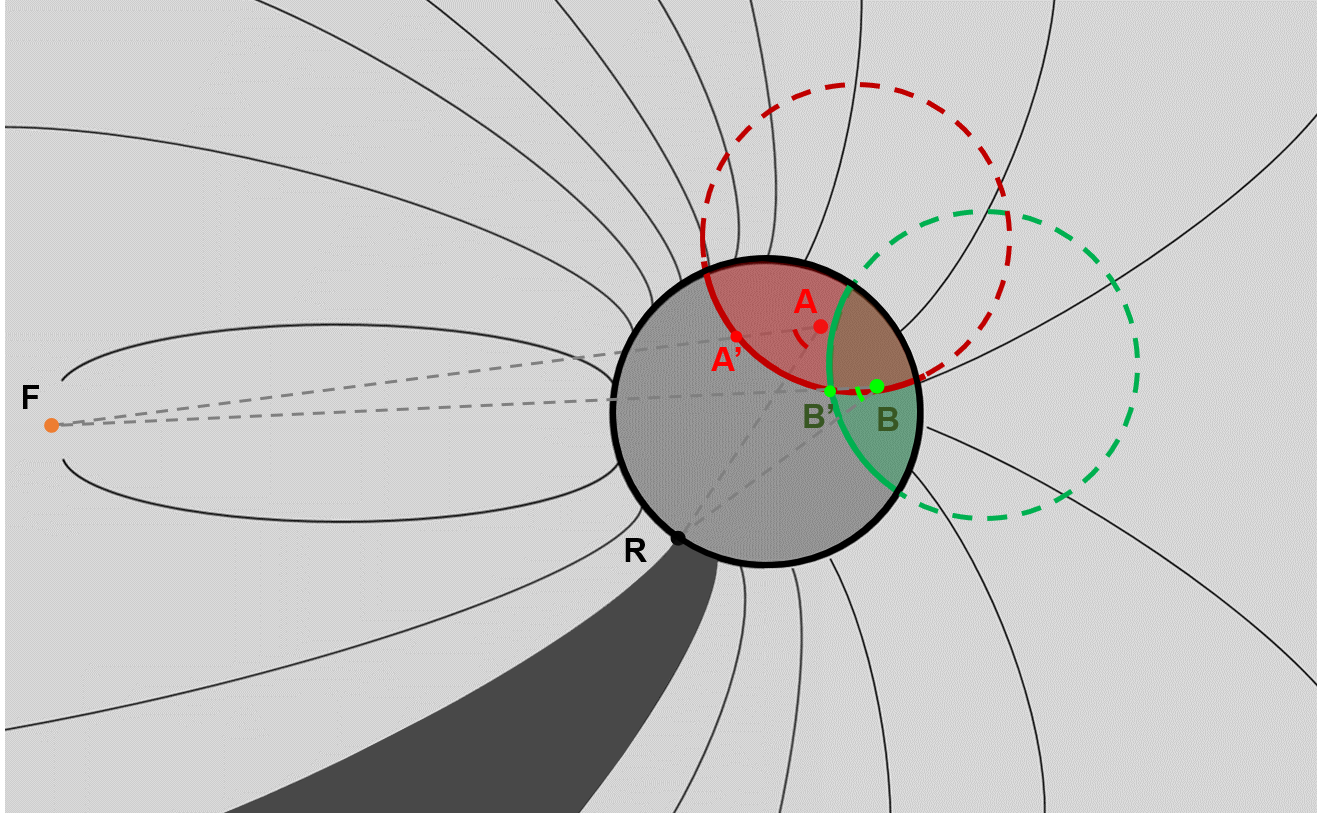

Supplement: Supplementary file 1 — Supplementary Figure 1. [file 41598_2024_58749_MOESM1_ESM.tif]
