# Supplementary material for: Optic nerve head factors associated with initial central visual field defect in primary open-angle glaucoma
Source: Sci Rep. 2024 Apr 5;14:8000. doi: 10.1038/s41598-024-58749-6 (PMC10997601; doi:10.1038/s41598-024-58749-6)
Supplement: Supplementary file 2 — Supplementary Legends. [file 41598_2024_58749_MOESM2_ESM.docx]

**Supplementary Figure 1.** Effect of lamina cribrosa (LC) and Bruch’s membrane opening (BMO) offset when evaluating retinal nerve fiber layer defect (RNFLD) proximity. The overlying retinal layer has an RNFLD (dark gray) and the BMO (gray circle with black border). The red dotted circle indicates more oblique LC/BMO offset, and the red ellipse indicates the funduscopic optic disc margin. The green dotted circle indicates less oblique LC/BMO offset, and the green ellipse indicates funduscopic optic disc margin. Reference lines (gray dotted lines) are draw from the fovea (F, orange dot) to each disc center (A and B). The cross-sectional point between the reference line and each optic disc margin is marked as A’ and B’, respectively. Please note that even though the location of the proximal margin of the RNFLD (R, black dot) was unchanged, the RNFLD proximity to the fovea was measured at a larger value in the case of oblique LC/BMO offset, due to the change of the disc center (A). Therefore, oblique LC/BMO offset might conceal the actual proximity of RNFLD when assessed from the funduscopic disc margin. In eyes with oblique LC/BMO offset, a greater disc-foveal angle and a shorter disc-margin-to-fovea distance might arise (FA’ < FB’).
